# The complete chloroplast genome sequence of *Juniperus saltuaria* (Cupressaceae)

**DOI:** 10.1080/23802359.2020.1781559

**Published:** 2020-06-24

**Authors:** Xinjun Zhang, Xingle Qu, Jiangrong Li, Jian Huang, Yuhuan Jia, Shujun Chen

**Affiliations:** a Res. Institute of Tibet Plateau Ecology, Tibet Agriculture and Animal Husbandry University, Nyingchi, China; b Key Laboratory of Forest Ecology in Tibet Plateau (Tibet Agriculture and Animal Husbandry University), Ministry of Education, Nyingchi, China; c College of Forestry, Northwest A&F University, Yangling, China

**Keywords:** *Juniperus saltuaria*, Illumina sequencing, chloroplast genome, phylogenetic analysis

## Abstract

In order to supply genetic information of *Juniperus saltuaria*, we reported the complete chloroplast genome sequence based on high-throughput sequencing data. The whole chloroplast genome was 128,099 bp long with an asymmetric base composition (32.9% A, 16.9% C, 18.1% G and 32.1% T). The genome annotation predicted a total of 116 genes, including 82 protein-coding genes, 30 tRNA genes, and 4 rRNA genes. The neighbor-joining phylogenetic analysis based on 45 complete chloroplast genome sequences showed that *J. saltuaria* was more closely related to the congeneric *J. recurva*. The assembled chloroplast genome of *J. saltuaria* will provide useful genomic data both for the phylogenetic research of *Juniperus* and the conservation of this species.

*Juniperus saltuaria*, an evergreen plant in the family Cupressaceae, is mainly distributed in the sunny or semi-sunny slope of high mountains or hills of the western Sichuan, northwest Yunnan and Tibet. It is characterized by light loving, cold tolerance, drought resistance, wind resistance, and is also an endemic timberline tree in the Qinghai-Tibetan Plateau, China. It occurs mainly on sunny slope of the Sergyemla (Sygera) Mountains in southeast Tibet (Liu et al. 2016). *J. saltuaria* grows along an altitudinal gradient from 4200 to 4520 m on the slope (Liu et al. 2011). It forms a natural altitudinal treeline around 4500 m (Chen et al. 2018), which has the world’s highest elevation treeline (Miehe et al. 2007). Because of slow growth of *Juniperus*, the forest of alpine timberlines very difficult to renew and recover effectively in a short time once it is destroyed, especially the timberlines of sunny slope. Alpine timberlines are ecotones highly sensitive to disturbances and environmental changes. So, *J. saltuaria* may be useful in ecological restoration of the high-altitude ecosystem (Liu and Luo 2011; Sheng et al. 2014; Liu et al. 2016).To facilitate its phylogenetic analysis and conservation genetics of this species and sustainable utilization, we assembled its complete chloroplast genome by Illumina sequencing technology in this study. The chloroplast genome sequence was submitted to GenBank (accession number MT133566).

Fresh leaves of *J. saltuaria* were collected from Mt. Sergyemla in Nyingchi (Linzhi) (29°39′N, 94°42′E), Tibet, China. A voucher specimen was deposited at the Herbarium of the Research Institute of Xizang Plateau Ecology (XZE), Tibet Agriculture & Animal Husbandry University. The genomic DNA was extracted using the DNeasy Plant Mini Kit (QIAGEN, Valencia, CA). High-throughput sequencing was conducted on the Illumina HiSeq platform. A total of 74,978,802 of 150 bp raw paired reads were generated, quality-trimmed with CLC Genomics Workbench v10 (CLC Bio, Aarhus, Denmark), and subjected to assembly of chloroplast genome with MITObim v1.9 (Hahn et al. 2013). The chloroplast genome of *Juniperus cedrus* (KT378453) (Guo et al. 2016) was selected as the initial references. Genome annotation was conducted in GENEIOUS R11 (Biomatters Ltd., Auckland, New Zealand) by aligning with those of phylogenetically related species. A physical map was drawn with the web-based tool OGDRAW (Lohse et al. 2013).

The whole chloroplast genome of *J. saltuaria* was determined to be 128,099 bp in length. As commonly found in chloroplast genomes of other higher plants, the base composition was asymmetric (32.9% A, 16.9% C, 18.1% G and 32.1% T) with an overall A + T content of 65.0%. It encodes a set of 116 genes, including 82 protein coding, 30 tRNA and 4 rRNA genes. Almost all genes occurred in a single copy with the exception of *trnQ-UUG* being duplicated. Among these genes, 15 genes harbored a single intron (*atpF, ndhA, ndhB, petB, petD, rpl2, rpl16, rpoC1, rps12, trnA-UGC, trnG-UCC, trnI-GAU, trnK-UUU, trnL-UAA* and *trnV-UAC*) and 1 genes (*ycf3*) harbored two introns.

To investigate phylogenetic status of *J. saltuaria*, a neighbor-joining phylogeny was reconstructed from the complete chloroplast genomes of 45 species within the order Cupressaceae using the concatenated sequences of chloroplast protein-coding genes with MEGA7 (Kumar et al. 2016) performed with 1000 replicates (Figure 1). The phylogenetic analysis indicated that *J. saltuaria* was more closely related to the congeneric *J. recurva* (Song et al. 2019). The complete chloroplast genome of *J. saltuaria* will supply useful genetic information for population genetic survey, conduct phylogenetic analysis, evolutionary studies, and conservation strategies for this valuable tree species.

**Figure 1.**
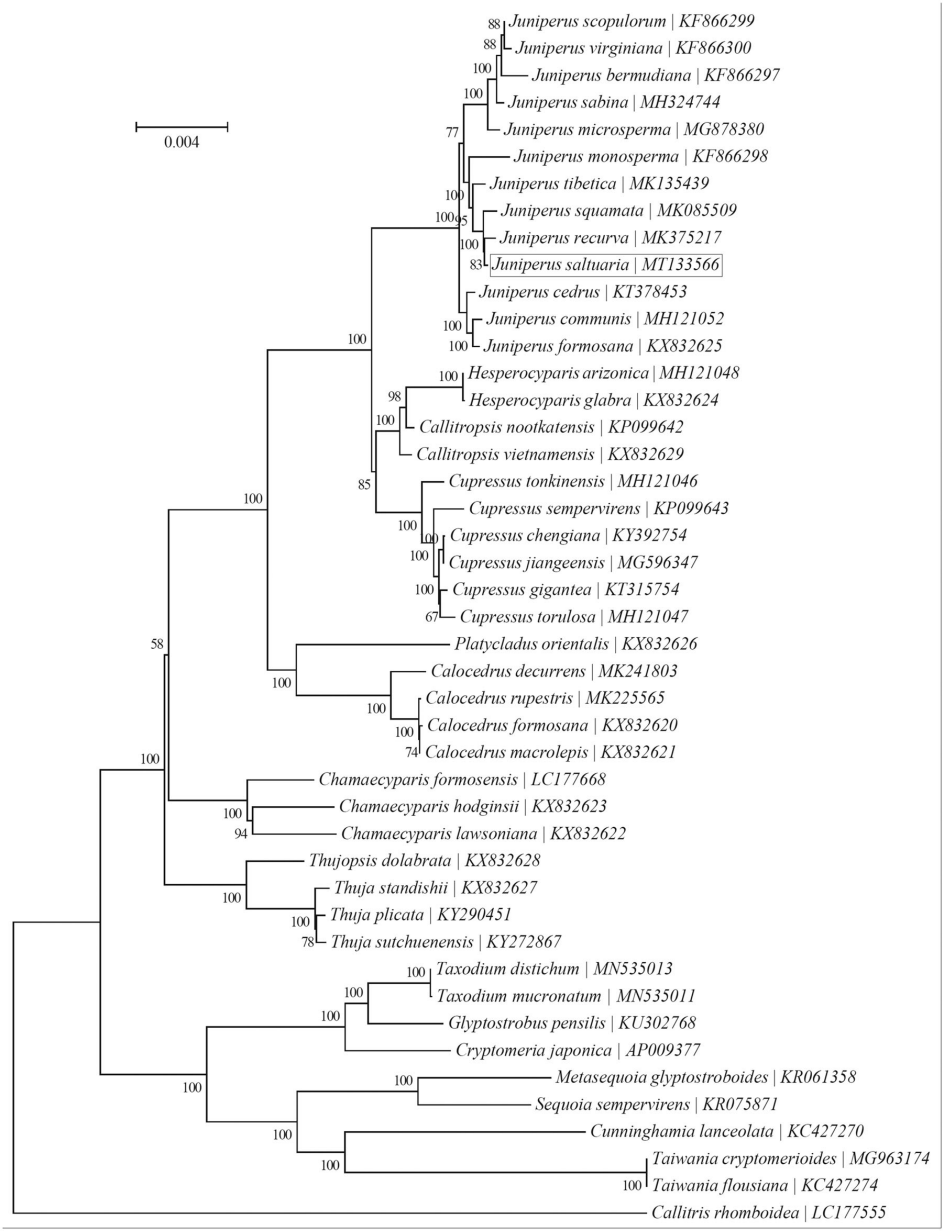
Phylogenetic relationships of 45 species based on the neighbor-joining analysis of chloroplast protein-coding genes. The bootstrap values were based on 1000 replicates, and are shown next to the branches.

## Data Availability

The data that support the findings of this study are openly available in GenBank of NCBI at https://www.ncbi.nlm.nih.gov, reference number MT133566.
